# Adipocyte-specific deletion of gp130 prevents ketogenic diet–induced hepatic steatosis

**DOI:** 10.1097/HC9.0000000000000782

**Published:** 2025-08-26

**Authors:** Berkay Senkalfa, Melanie Gloor, Ronja Podlaszewski, Revati S. Dewal, Carla Horvath, Vissarion Efthymiou, Adhideb Ghosh, Stephan Wueest, Daniel Konrad, Christian Wolfrum, Tenagne D. Challa

**Affiliations:** 1 Institute of Food Nutrition and Health,Department of Health Sciences and Technology, Eidgenössische Technische Hochschule Zürich Schwerzenbach, Switzerland; 2 Division of Pediatric Endocrinology and Diabetology, University Children’s Hospital, University of Zurich, Zurich, Switzerland; 3 Children’s Research Center, University Children’s Hospital, University of Zurich, Zurich, Switzerland; 4 Zurich Center for Integrative Human Physiology, University of Zurich, Zurich, Switzerland

**Keywords:** gp130, HSL, IL-6, JNK, ketogenic diet, lipolysis, MASH, MASLD, p38, WAT

## Abstract

**Background:**

Metabolic dysfunction–associated steatotic liver disease (MASLD), the hepatic manifestation of obesity and type 2 diabetes, can progress to metabolic dysfunction–associated steatohepatitis and fibrosis. MASLD is characterized by elevated hepatic lipid accumulation (steatosis) and insulin resistance. The ketogenic diet (KD), a high-fat, low-carbohydrate diet, induces hepatic insulin resistance and steatosis in animal models through unknown mechanisms.

**Methods and Results:**

Herein, we investigated the mechanisms behind KD-induced metabolic dysfunction–associated steatohepatitis and fibrosis at thermoneutrality, identifying upregulated inflammatory and lipogenic pathways, including *Il-6*, *Tnf*, *Mapk13*, *Lpl*, and *Pparg*. Given the substantial increase in IL-6 during MASLD progression, we investigated IL-6-gp130 signaling using liver- and adipocyte-specific knockout mice. Liver-specific gp130 deletion failed to prevent KD-induced hepatic steatosis and glucose intolerance. In contrast, adipocyte-specific gp130 deletion significantly reduced KD-induced hepatic steatosis by suppressing lipolysis in white adipose tissue and reducing p-JNK and p-p38 signaling in the liver. In agreement, adipocyte-specific deletion of gp130 protected mice from KD-induced hepatic steatosis in response to recombinant IL-6 treatment.

**Conclusions:**

Our studies demonstrate the importance of adipose tissue-liver crosstalk in mediating MASLD progression and identify adipocyte IL-6-gp130 as a potential therapeutic target.

## Graphical abstract

**Figure:**
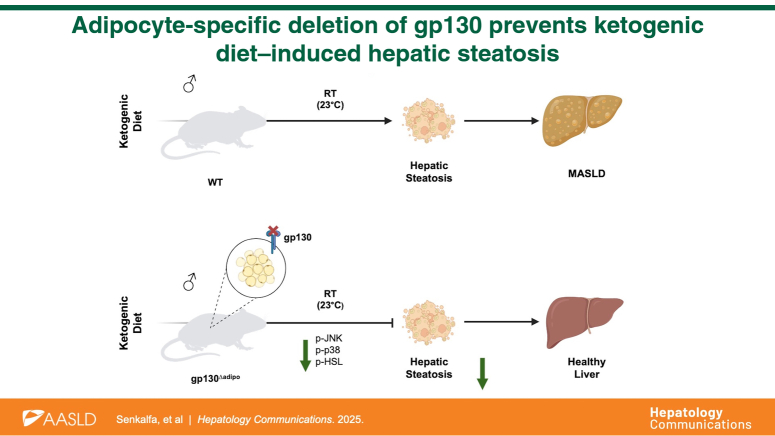

## INTRODUCTION

Metabolic dysfunction–associated steatotic liver disease (MASLD), formerly known as metabolic dysfunction–associated fatty liver disease, is a hepatic manifestation of metabolic disorders associated with obesity and type 2 diabetes. MASLD is becoming a global pandemic^1^ and can progress from hepatic steatosis to metabolic dysfunction–associated steatohepatitis (MASH), liver fibrosis, HCC, and end-stage liver disease.^2^,^3^ MASH, characterized by inflammation and liver damage,^4^,^5^ is linked to cardiovascular diseases^6^ and increases mortality rates.^7^ MASLD affects over a billion adults in high-income countries^8^ and is the second leading cause of liver transplants due to a lack of approved therapies, with annual costs reaching $100 billion in the United States^7^ and exceeding €235 billion in Europe.^9^ Liver transplantation leads to a high mortality rate due to reduced tissue regeneration and increased transplant intolerance, indicating the urgent need for alternative therapies.^10^ MASLD is mainly caused by obesity and a genetic predisposition that leads to energy imbalance and liver dysfunction.^11^ However, lean individuals can also develop MASLD, which is more prevalent in males but causes more liver damage in females.^12^ Mechanistically, increased hepatic de novo lipogenesis, increased lipolysis in adipose tissue, decreased fat oxidation, and/or reduced hepatic VLDL secretion can contribute to MASLD.^13^,^14^,^15^ Furthermore, in obesity, adipose-derived cytokines like IL-6, TNFα, and IL-1β activate and recruit macrophages to the liver, promoting liver inflammation, hepatocyte damage, and fibrosis.^16^,^17^

The IL-6 family cytokines signal through the gp130 receptor through classical, trans, or cluster pathways.^18^ IL-6 acts as a pleiotropic cytokine with various physiological and pathophysiological functions, and its effects depend on the source.^19^ For example, IL-6 released from skeletal muscle during exercise^20^ increases glucagon-like peptide 1, which improves glucose homeostasis in mice^21^ and humans.^22^,^23^ In contrast, IL-6 released from adipose tissue enhances free fatty acid (FFA) secretion through gp130, promoting diet-induced hepatic steatosis and insulin resistance in obese mice.^24^,^25^ In particular, adipocyte-specific gp130 knockout (AT-gp130 KO) decreased high-fat diet (HFD)-induced lipolysis in mesenteric adipocytes, thereby blunting the development of hepatic steatosis and insulin resistance in mice.^25^ Clinical studies of gp130 signaling, such as engineered gp130 cytokine, IC7Fc, have shown promising results in treating obesity, hepatic steatosis, and type 2 diabetes.^26^

The ketogenic diet (KD), a high-fat, low-carbohydrate diet, was initially developed for epilepsy treatment and has gained popularity for its benefits in aiding weight loss and improving insulin sensitivity in obese humans.^3^,^5^,^27^ Ketogenesis might be associated with decreased risk of MASLD in type 2 diabetes.^28^ KD-induced body weight reduction, accompanied by elevated GDF15 levels, is absent in GDF15 or GFRAL-deficient mice, highlighting the crucial role of GDF15-GFRAL signaling in KD-mediated weight loss.^28^ Clinical studies comparing the Mediterranean diet to the very low-calorie KD reported weight loss in both regimens, with the Mediterranean diet leading to better outcomes in waist circumference after 3 months and 1-month feedings, respectively.^29^

In 10 obese subjects, 6 days of KD feeding reduced intrahepatic triglycerides, body weight, and hepatic insulin resistance despite increased FFA concentrations due to an enhanced hepatic mitochondrial redox state.^30^ In contrast, 3 days of KD induced more pronounced hepatic insulin resistance in mice versus HFD-feeding, as revealed by impaired insulin-mediated suppression of hepatic glucose production despite normal glucose uptake in peripheral tissues.^31^ We also showed that KD-fed mice for 14 weeks at thermoneutrality (TN) developed MASH and fibrosis, but not at room temperature (RT).^32^ Thermoneutral housing reduces energy expenditure and alters immune and lipid metabolism, thereby promoting MASLD in HFD-fed mice.^33^ In the current study, we further investigate the mechanisms behind MASH and fibrosis formation in KD-fed C57BL/6 mice at TN compared with RT. Moreover, we aim to investigate the role of IL-6-gp130 signaling in KD-induced hepatic steatosis and glucose intolerance using liver and adipocyte-specific gp130 knockout mice.

## METHODS

### Animals

Adipocyte-specific (gp130^∆adipo^) and liver-specific (gp130^∆alb^) gp130 knockout mice on a C57BL/6J background were generated by crossing gp130 floxed (gp130^F/F^) mice to animals expressing the Cre recombinase (*Cre*) controlled by the adiponectin (*Adipoq*) or albumin (*Alb*) promoter, respectively. As controls, littermate mice that do not express *Cre* were used (gp130^F/F^). All animals were housed in a pathogen-free animal facility either at ambient RT (23°C) or at TN (30°C) under a 12-hour light/dark cycle with free access to water and standard chow diet (18% proteins, 4.5% fibers, 4.5% fat, and 6.3% ashes of energy, #2222, Kliba-Nafag). After 12 weeks of chow, male mice were fed either a chow, a KD (8.5% protein, 4.3% fibers, 79.1% fat, 4.3% ashes, and 3.8% carbohydrate of calories, E15149 Snniff), or an HFD with 60% of energy derived from fat (23.9% protein, 3% fibers, 35% fat, 5.7% ashes, and 23.2% carbohydrate, #3436, Kliba-Nafag). All animal studies conformed to the Swiss animal protection laws and were approved by the cantonal Veterinary Office in Zurich, Switzerland. Experimental animals were randomly assigned to either a chow, KD, or HFD for short term (3 d) or long term (14 wk).

All experiments were performed using male C57BL/6J mice, as female mice exhibit estrogen-mediated metabolic protection, making them less susceptible to diet-induced metabolic changes).^1^,^2^ This sex-based difference is well-documented in metabolic studies, as estrogen regulates adipose tissue metabolism and inflammatory responses, leading to lower hepatic lipid accumulation and improved insulin sensitivity in females compared with males.

#### Mouse strain validation

To assess potential differences in metabolic responses between C57BL/6J and C57BL/6N mice, we compared body weight changes under HFD conditions. Male C57BL/6J and C57BL/6N mice were fed an HFD for 16 weeks, followed by daily body weight measurements over 28 days (*data not shown*). Both strains exhibited comparable weight gain in response to HFD-feeding, indicating similar metabolic responses. Based on these observations, C57BL/6N mice were used for the thermoneutral gene expression profiling experiments, while all genetic experiments were performed in C57BL/6J mice.

### Genotyping

Mice were genotyped using PCR for floxed alleles and qPCR for the Cre transgene. Primer details are available on request.

### Recombinant IL-6 administration

Recombinant mouse IL-6 (Catalog #406-ML-005, R&D Systems) was reconstituted in sterile PBS containing 0.1% BSA to a final concentration of 10 µg/mL. For injection, 2.5 µL of IL-6 stock was diluted in 97.5 µL of sterile PBS to deliver a 1 mg/kg/d dose. Twelve-week-old male gp130^F/F^ and gp130^∆adipo^ mice were injected i.p. with IL-6 (100 µL per mouse) or vehicle (PBS + 0.1% BSA) once daily for 3 consecutive days during KD feeding.

### Protein extraction and western blotting

All samples were lysed in cold RIPA buffer (50 mM Tris-HCl pH 7.4, 150 mM NaCl, 2 mM EDTA, 1.0% Triton X100, and 0.5% sodium deoxycholate) containing protease (Complete, Roche) and phosphatase (Thermo Fisher) inhibitors.^32^ The samples were centrifuged at 24,000*g* for 10 minutes at 4°C, and protein concentrations were measured by using Pierce BCA Protein assay Kit (#23225, Thermo Fisher Scientific).^34^ Equal amounts of protein (20–60 μg) were resolved on (8%–15%) an SDS-polyacrylamide gel and transferred onto a nitrocellulose membrane (#1620112, Bio-Rad) as described in the supplemental sections, http://links.lww.com/HC9/C69.

### RNA extraction and quantitative real-time PCR

Total RNA was extracted from the livers of mice fed chow, KD, or HFD for 14 weeks, using Trizol reagent (#15596026, Thermo Fisher) according to the manufacturer’s protocol. Genomic DNA was treated with DNase 10X DNase I buffer and DNase (1:4000, M0303L, NEB BioLabs) at 37°C for 20 minutes. Reverse transcription was performed using the High-Capacity cDNA Reverse transcription kit (#4368814, Applied Biosystems) with 0.2 μg–1 μg of the total RNA. Quantitative PCR was performed with the KAPA SYBR FAST qPCR master mix (2X) kit (Roche, KK4602) using a concentration of 5 ng/μL to yield 10 ng/well of cDNA, on a ViiA7 (Applied Biosystems). Relative mRNA concentrations normalized to the expression of the housekeeping gene 36B4 were calculated using the ^ΔΔ^Ct method. Primers will be provided on request.

### RNA sequencing

For RNA sequencing, mice were fed chow, KD, or HFD for 14 weeks at RT or TN. Total RNA was extracted using Trizol reagent (#15596026, Thermo Fisher) according to the manufacturer’s protocol. Genomic DNA was treated with DNase 10X DNase I buffer and DNase (1:4000, M0303L, NEB BioLabs) at 37°C for 20 minutes. RNA sequencing was performed at Novogene. The quality of the isolated RNA was determined with a Fragment Analyzer (Agilent). RNA sequencing libraries were prepared using Illumina TruSeq Stranded mRNA (Illumina, Inc.). Sequencing was performed as 50 bp, single reads, and 7-base index reads on an Illumina HiSeq2000 instrument as described.^9^ The raw reads were first cleaned by removing adapter sequences, trimming low-quality ends, and filtering reads with low quality (phred quality <20) using Trimmomatic (Version 0.36).^8^ Sequence pseudo alignment of the resulting high-quality reads to the Mouse reference genome (build GRCm38.p6) and quantification of gene-level expression (gene model definitions based on GENCODE release M23) was carried out using Kallisto (Version 0.44.0).^9^ Differential expressions were computed using the generalized linear model implemented in the Bioconductor package edgeR (R version: 3.6.1, edgeR version: 3.28.0) [edgeR]. Genes showing altered expression with adjusted (Benjamini and Hochberg method) *p* value<0.05 were considered differentially expressed.^9^ We generated a heatmap of differentially expressed genes at a threshold of *p*<0.01, FDR <0.032, log2 ratio >0.5, and fold change of log2 ratio >0.5 for upregulated genes, and of *p*<0.01, FDR <0.032, log2 ratio ≤ 0.5, and fold change of log2 ratio ≤ 0.5 for downregulated genes. The same thresholds were employed for principal component analysis and volcano plots. The data were obtained and analyzed using SUSHI, a genomic data management and analysis platform of the Functional Genomics Center Zürich. Data are presented as the regularized (r) log of the counts.

### Quantification and statistical analysis

A power calculation was performed based on the results of previous work by our group to calculate animal numbers.^31^ All data are expressed as mean ± SEM. The significance was determined using a 2-tailed, unpaired Student’s *t* test, 1-way ANOVA with Newman-Keuls correction for multiple group comparisons, and 2-way ANOVA with Bonferroni multiple comparisons/Tukey’s multiple comparison. Statistical tests were calculated using GraphPad Prism 10.3.1 (GraphPad Software). *p* values<0.05 were considered significant.

### Data and code availability

Bulk RNAseq data are available in GEO under the accession number *GSE294746*. The original data for the uncropped and unmodified images in this paper are available upon request. For further methodologies employed in this study described in detail, please refer to the Supplemental Manuscript, http://links.lww.com/HC9/C69.

## RESULTS

### KD-fed mice develop MASH and fibrosis at TN but not at RT

**FIGURE 1:**
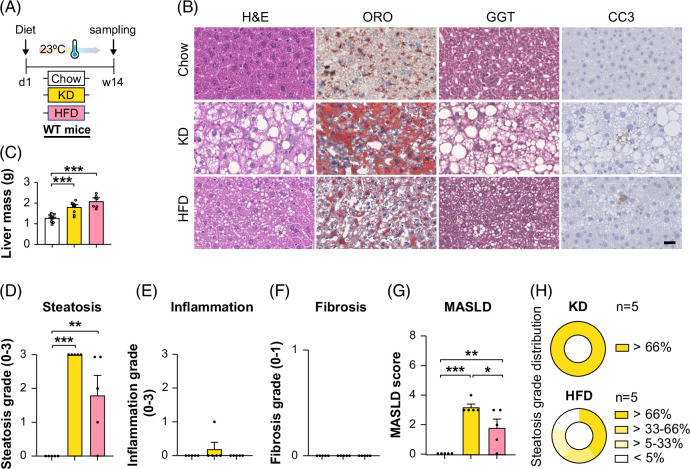
KD-fed C57BL/6 mice develop MASH and fibrosis at thermoneutrality but not at room temperature. (A) Experimental scheme for chow, HFD, or KD feeding mice for 14 weeks at room temperature, fasted for 6 hours before sampling. (B) Representative images of liver sections stained with H&E, oil red O, GGT (for fibrosis), and cleaved caspase 3 (CC3); scale bar represents 100 μm. (C) Liver mass. Grades of histopathological MASH: (D) steatosis grade, (E) lobular inflammation, (F) fibrosis scores, (G) total MASLD score, (H) steatosis distribution score, n=5 per group. Values are presented as mean ± SEM. *p<0.05, **p<0.01, ***p<0.001, by 1-way ANOVA + Tukey’s multiple comparisons. Abbreviations: HFD, high-fat diet; KD, ketogenic diet; MASH, metabolic dysfunction–associated steatohepatitis.

**FIGURE 2:**
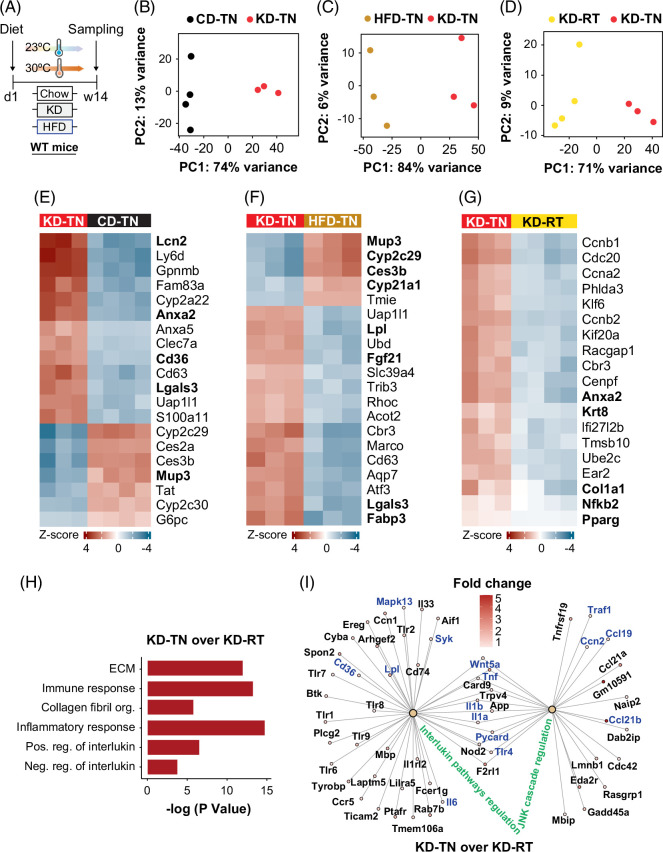
KD upregulates inflammatory pathways and JNK signaling cascades, leading to MASH and fibrosis. (A) Experimental scheme for chow, HFD, or KD feeding C57BL/6N mice at TN or RT for 14 weeks, fasted for 6 hours before sampling. RNAseq was performed on the liver of mice (n=4 per group). (B) PCA of chow (CD)-TN versus KD-TN, (C) HFD-TN versus KD-TN, (D) KD-RT versus KD-TN. (E–G) Heatmap of differentially expressed genes. (H) The biological process analysis in KD-TN versus KD-RT. (I) Network analysis in KD-TN versus KD-RT. Differentially expressed genes were analyzed at the threshold of *p* <0.01, log2 ratio >0.5, and fold change of log2 ratio >0.5 and presented as the regularized (r) log of the counts. Abbreviations: HFD, high-fat diet; KD, ketogenic diet; MASH, metabolic dysfunction–associated steatohepatitis; PCA, principal component analysis; RNAseq, RNA sequencing; RT, room temperature; TN, thermoneutrality.

Recently, we reported that KD-fed C57BL/6 mice at TN (30°C, TN) exhibit aggravated hepatic steatosis, inflammation, fibrosis, MASH, and MASLD compared with HFD-fed mice.^32^ In the current study, 14 weeks of KD feeding at RT (23°C) similarly induced severe hepatic steatosis, inflammation, and MASLD but not liver fibrosis or MASH (Figures 1A–H). To investigate the mechanism by which KD induces liver fibrosis and MASH at TN, we analyzed liver RNAseq data from wild-type mice fed chow (CD), KD, or HFD for 14 weeks, exposed to RT or TN (Figure 2A). Principal component analysis demonstrated a significant separation of genes under KD compared with other diets at TN (Figures 2B–D). KD-TN versus KD-RT revealed a distinct separation, with a PC1 variance of 71% (Figure 2D), indicating substantial gene expression differences between TN and RT. Heatmap and volcano plots revealed upregulated *Lcn2*, *Anxa2*, *Cd36*, *Lgals3*, and downregulated *Mup3* in KD-TN versus CD-TN, suggesting increased lipid uptake (Supplemental Figure S1A, http://links.lww.com/HC9/C69, Figure 2E). In KD-TN versus HFD-TN, genes involved in lipid metabolism, including *Fgf21* and *Fabp3*, were upregulated, suggesting metabolic shifts to counteract lipid accumulation. Conversely, *Mup3*, *Cyp2c29*, *Ces3b*, and *Cyp21a1* were downregulated, indicating impaired lipid processing (Supplemental Figure S1B, http://links.lww.com/HC9/C69, Figure 2F).

Increased *Lpl* expression in KD-TN may contribute to elevated liver lipid accumulation compared with HFD-TN (Figure 2F). Similarly, *Pparg* upregulation in KD-TN versus KD-RT suggests enhanced lipid uptake and triglyceride storage, contributing to steatosis (Figure 2G). Furthermore, KD-TN elevated *Col1a1* and *Anxa2*, promoting ECM depositions that may initiate fibrosis, alongside increased *Nfkb2* and *Krt8* expression, accelerating inflammation and apoptosis, key steps in the progression of steatosis to MASH (Supplemental Figure S1C, http://links.lww.com/HC9/C69, Figure 2G).

Biological process analyses identified upregulated inflammatory response, immune response, and ECM organization in KD-TN versus KD-RT (Figure 2H). Supporting these findings, network analyses showed significantly upregulated genes involved in inflammatory responses and JNK signaling cascades in KD-TN versus KD-RT (Figure 2I). For instance, *Tnf, IL-6, IL-1b, Ccl21, Mapk13, Tlr4,* and *Traf1* were significantly upregulated in KD-TN, accelerating inflammation. In addition, *Cd36* and *Lpl* upregulation in KD-TN versus KD-RT (Figure 2I) may enhance lipid storage, further contributing to hepatic steatosis and metabolic stress.

In CD-TN versus CD-RT, downregulated *Ehhadh, Pdk4,* and *Cpt1b* suggest impaired fatty acid oxidation at TN under chow (Supplemental Figures S1D, E, http://links.lww.com/HC9/C69). In addition, *Cidea* was significantly upregulated in HFD-TN versus HFD-RT (Supplemental Figure S1J, http://links.lww.com/HC9/C69), indicating accelerated hepatic steatosis in HFD-fed mice at TN and supported by the observed histological data.

Heatmap and volcano plots also show distinct gene expression changes in KD-RT versus CD-RT. For instance, *Ccnd1* is upregulated in KD-RT, while *Scd1*, *Gstm2*, and *Cyp2c29,* genes that regulate lipid metabolism, are downregulated in KD-RT (Supplemental Figures S1F–H, http://links.lww.com/HC9/C69). Similarly, *Ccnd1*, *Serpine2*, and *Mup3* were upregulated in KD-RT compared with HFD-RT, while *Scd1* and *Cyp2c70* were downregulated (Supplemental Figure S1G–I, http://links.lww.com/HC9/C69).

Our findings demonstrate that inflammatory and metabolic stress pathways, including TNF, IL-6, IL-1β, and MAPK13 signaling, promote the progression of hepatic steatosis to MASH and fibrosis under KD at TN but not at RT. Altogether, our data suggest that targeting IL-6 and JNK-MAPK signaling could be a promising therapeutic strategy for MASH and fibrosis.

### Expression of lipolytic and lipogenic pathways in KD-fed C57BL/6N mice

We performed RT-PCR to confirm the upregulation of genes identified in RNAseq analysis in the livers of C57BL/6N mice after 14 weeks on chow, KD, or HFD at RT (Supplemental Figure S2A, http://links.lww.com/HC9/C69). *Lpl* mRNA was significantly increased in KD compared with chow, while *Mgl* levels decreased, and *Atgl* levels were unchanged between chow and KD (Supplemental Figures S2B–D, http://links.lww.com/HC9/C69). We assessed mRNA levels of genes involved in TG synthesis and found increased *Mgat3* expression in KD-fed mice, while *Dgat1, Dgat2*, and *Dgkd* were significantly reduced (Supplemental Figures S2E–H, http://links.lww.com/HC9/C69). *Lpin1*, but not *Lpin2*, expression was decreased in KD-fed mice (Supplemental Figures S2I, J, http://links.lww.com/HC9/C69). Among phospholipid markers, *Plpp2* increased in KD-fed mice, while *Ppara* and *Pgc1a*, regulators of beta-oxidation, were reduced (Supplemental Figures S2K–M, http://links.lww.com/HC9/C69). Unlike *Pparg*, which can exacerbate MASH progression, knockouts of *Ppara* are linked to MASLD development, while *Pgc1a* overexpression reduces hepatic lipid accumulation.^35^,^36^,^37^ Thus, KD-driven downregulation of *Ppara* and *Pgc1a* highlights their roles in hepatic steatosis and MASLD progression. *Ept1* and *Cept1* showed no differences between dietary groups, whereas *Cds2* was reduced in both KD and HFD-fed groups (Supplemental Figures S2N, O, http://links.lww.com/HC9/C69). Upregulated *Lpl* and downregulated *Ppara* and *Pgc1*a can indicate the metabolic impact of KD on liver lipid metabolism. Collectively, our mRNA data corroborate the RNAseq findings, confirming that KD induces more severe hepatic steatosis than HFD.

### Liver-specific gp130 deletion does not protect from KD-induced hepatic steatosis

**FIGURE 3:**
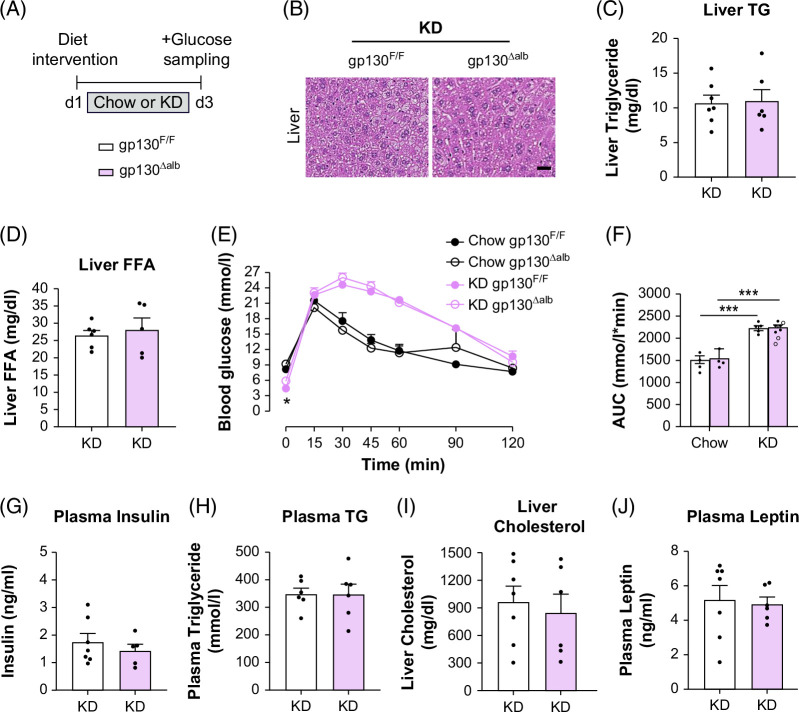
Liver-specific gp130 deletion does not protect mice from KD-induced glucose intolerance and hepatic steatosis. (A) Experimental scheme for chow or KD feeding in WT (gp130^F/F^) and liver gp130 KO (gp130^∆alb^) mice for 3 days, fasted for 6 hours before sampling. (B) Representative images of liver sections stained with H&E from KD-fed mice; scale bar is 100 μm. (C) Liver TG content (D), liver-FFA (n=6-7 per group). (E) I.p. glucose-tolerance test (i.p.GTT) and (F) AUC in chow or KD-fed gp130^∆alb^ and gp130^F/F^ mice for 3 days (chow n=4, KD n=6 per group). (G) plasma insulin, (H) plasma TG, (I) liver cholesterol, and (J) plasma leptin (n=6–7 per group). Values are presented as mean ± SEM. ***p<0.001 by 2-way ANOVA (E, F), Student *t* test for C, D, G–J. Abbreviations: FFA, free fatty acid; KD, ketogenic diet; TG, triglyceride.

Given that RNAseq analysis identified upregulated IL-6 and JNK signaling in KD-induced MASH and fibrosis, we investigated whether liver IL-6-gp130 disruption could alleviate KD-induced MASLD and insulin resistance in liver-specific gp130 knockout (gp130^∆alb^) mice and littermate controls (gp130^F/F^), generated using the Cre-lox system. Gene expression analysis confirmed the deletion of gp130 mRNA in the liver of gp130^∆alb^ mice, while its expression remained comparable between gp130^∆alb^ and gp130^F/F^ mice in brown and white adipose tissue depots (Supplemental Figure S3A, http://links.lww.com/HC9/C69). We observed similar body weights in gp130^F/F^ and gp130^∆alb^ fed a chow or KD for 3 days (Figure 3A; Supplemental Figure S3B, http://links.lww.com/HC9/C69). Hepatic lipid droplet accumulation, liver triglyceride (TG) content, and liver-FFA levels were similar in both genotypes fed KD for 3 days (Figures 3B–D). KD significantly reduced fasting blood glucose levels but impaired glucose tolerance in gp130^∆alb^ and gp130^F/F^ mice (Figures 3E, F), indicating that liver gp130 deletion does not prevent KD-induced glucose intolerance. Plasma insulin, TG, leptin, and liver cholesterol levels were similar in KD-fed mice regardless of genotype (Figures 3G–J). To investigate whether liver gp130 deletion signals to the adipose tissue, we performed western blot analyses and showed no difference in ATGL, p-HSL, perilipin, p-JNK, and p-AKT protein levels in epididymal white adipose tissue (epiWAT) and mesenteric white adipose tissue in KD-fed gp130^∆alb^ and gp130^F/F^ mice (Supplemental Figures S3C–N, http://links.lww.com/HC9/C69). Altogether, liver gp130 deletion does not protect mice from KD-induced glucose intolerance and hepatic steatosis.

### Adipocyte-specific gp130 deletion prevents KD-induced hepatic steatosis but not glucose intolerance

**FIGURE 4:**
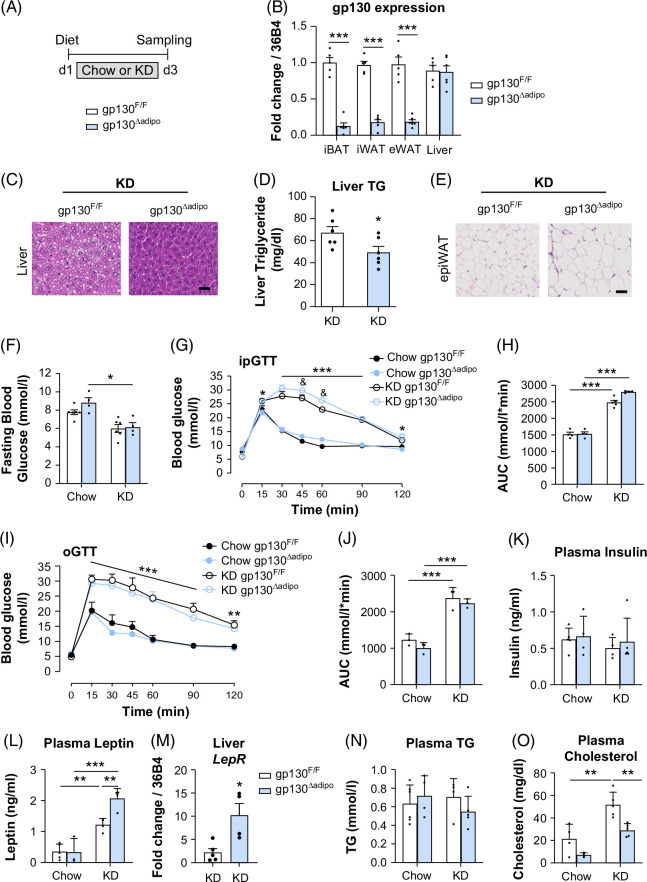
Adipocyte-specific gp130 deletion prevents KD-induced hepatic steatosis without affecting glucose intolerance. (A) Experimental scheme for chow or KD feeding gp130^F/F^ (WT) and adipose gp130^∆adipo^ (KO) mice for 3 days and fasted for 6 hours before sampling. (B) Gp130 expression in iBAT, ingWAT, epiWAT, and liver, n=5–6 per group. (C) Representative images of liver sections stained with H&E from KD-fed gp130^F/F^ and gp130^∆adipo^ mice; scale bar represents 100 μm. (D) Liver TG content (n=6 per group). (E) Representative images of epiWAT sections stained with H&E from KD-fed gp130^F/F^ and gp130^∆adipo^ mice; scale bar represents 100 μm. (F) Fasting blood glucose levels, (G) i.p.GTT, and (H) AUC in mice fed chow or KD (chow WT n=6; chow KO n=4; KD WT n=6; KD KO n=4). (I) oGTT and (J) AUC in chow or KD-fed gp130^∆adipo^ and gp130^F/F^ mice (n=3; in each group). (K) plasma insulin, (L) plasma leptin, (M) leptin receptor (*Lepr*) mRNA expression. (N) Plasma TG, (O) plasma cholesterol (n=4–5 per group). All values are expressed as mean ± SEM. *p<0.05; **p<0.01, ***p<0.001, by Student *t* test for B, D; 1-way ANOVA + Tukey’s multiple comparisons for F, H, J, K, L, M, N; 2-way ANOVA + Tukey’s multiple comparisons for G, I. Abbreviations: epiWAT, epididymal white adipose tissue; iBAT, interscapular brown adipose tissue; ingWAT, inguinal white adipose tissue; KD, ketogenic diet; oGTT, oral (o) glucose-tolerance test; TG, triglyceride.

Next, experiments were performed in adipocyte-specific gp130 knockout (gp130^∆adipo^) mice fed a standard chow or KD for 3 days (Figure 4A). As expected, gp130 expression was significantly reduced in interscapular brown adipose tissue, inguinal white adipose tissue, and epiWAT in gp130^∆adipo^ compared with control littermate (gp130^F/F^) mice, whereas similar gp130 mRNA levels were observed in the liver of both genotypes (Figure 4B). Importantly, the ablation of gp130 in adipocytes reduced hepatic lipid droplet accumulation and liver TG content (Figures 4C-D), revealing blunted KD-induced hepatic steatosis compared with controls. Moreover, KD-fed gp130^∆adipo^ mice exhibited larger adipocyte size in epiWAT compared with control littermates (Figure 4E), suggesting a pronounced hypertrophic response in adipocytes of the KO mice. Subsequently, i.p. and oral glucose-tolerance tests were performed to assess whether adipocyte-specific gp130 depletion affects glucose metabolism. While KD-fed mice exhibited lower fasting blood glucose levels (Figure 4F), i.p. and oral glucose tolerance were significantly impaired in both gp130^∆adipo^ and gp130^F/F^ mice after 3 days of KD feeding (Figures 4G–J), indicating that adipocyte-specific gp130 deletion does not prevent KD-induced glucose intolerance. Plasma insulin levels were unchanged regardless of diet and genotypes (Figure 4K). In line, insulin tolerance tests did not show a distinct phenotype between genotypes (Supplemental Figures S4A, B, http://links.lww.com/HC9/C69). Systemic leptin levels were significantly elevated in KD-fed gp130^∆adipo^ and gp130^F/F^ mice versus chow, with even higher levels observed in the KD-fed KO mice (Figure 4L), correlating with increased adipocyte size (Figure 4E). In agreement, liver leptin receptor expression was significantly increased in KD-fed gp130^∆adipo^ mice, suggesting leptin-mediated effects in the crosstalk between the liver and adipose tissues (Figure 4M). Systemic TG levels were similar between genotypes under KD and chow diets (Figure 4N), and plasma cholesterol levels were lower in KD-fed gp130^∆adipo^ mice than in control mice (Figure 4O). Collectively, adipocyte-specific gp130 deletion protected mice from KD-induced hepatic steatosis but not glucose intolerance.

### Adipocyte-specific gp130 deletion impairs epiWAT lipolysis and reduces liver JNK signaling under KD

**FIGURE 5:**
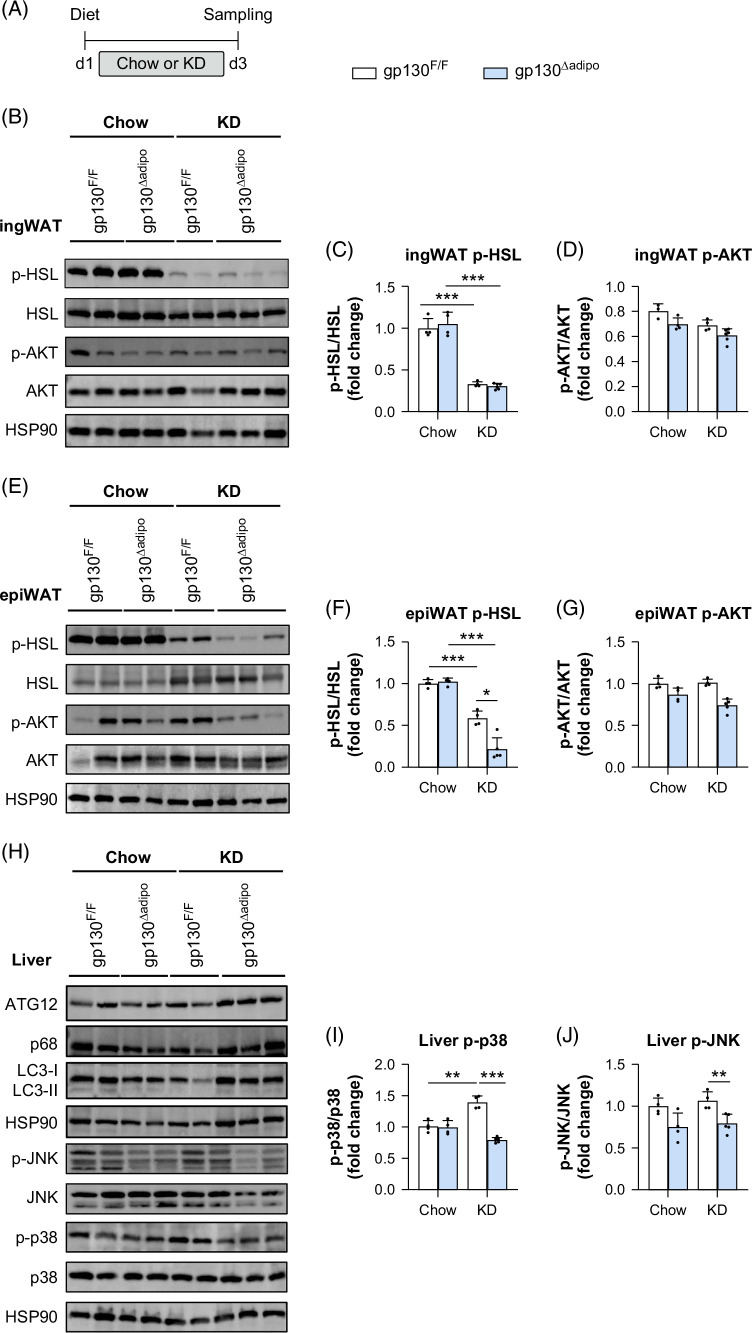
KD-fed gp130 adipocyte deletion impaired p-HSL protein levels and stress kinase. (A) Experimental scheme for chow or KD feeding gp130^F/F^ (WT) and gp130^∆adipo^ (KO) mice for 3 days and fasted for 6 hours before sampling. (B–D) Representative western blot analyses of p-HSL, and p-AKT protein expression and quantification in ingWAT (n=4–6 per group). (E–G) Representative western blot analyses of p-HSL, and p-AKT protein expression and quantification in epiWAT (n=4–6 per group). (H–J) Representative western blots analyses of p-JNK, p-p38, ATG12, p68, and LC3I/II protein expression and quantification in the liver (n=4–6 per group). All values are expressed as mean ± SEM. *p<0.05; **p<0.01, ***p<0.001, by 1-way ANOVA + Tukey’s multiple comparisons. Abbreviations: epiWAT, epididymal white adipose tissue; ingWAT, inguinal white adipose tissue; KD, ketogenic diet.

Despite protection from KD-induced hepatic steatosis, adipocyte-specific gp130 KO mice remained glucose-intolerant. To uncover these contrasting mechanisms, we investigated lipolytic and insulin signaling pathways after 3 days of KD or chow feeding, with or without glucose injection before dissection (Supplemental Figure S5A, http://links.lww.com/HC9/C69, Figure 5A). In glucose-injected mice, ATGL, p-HSL, perilipin-1, and p-AKT protein levels were unchanged in epiWAT (Supplemental Figures S5B–E, G, http://links.lww.com/HC9/C69) and mesenteric white adipose tissue (Supplemental Figures S5H–K, M, http://links.lww.com/HC9/C69) of KD-fed gp130^∆adipo^ and gp130^F/F^ mice. In contrast, p-JNK levels were significantly reduced in epiWAT of KD-fed gp130^∆adipo^ mice compared with controls (Supplemental Figure S5F, http://links.lww.com/HC9/C69) and remained unchanged in mesenteric white adipose tissue (Supplemental Figure S5L, http://links.lww.com/HC9/C69). Similar ATGL, p-HSL, and p-AKT protein levels were also observed in the livers of KD-fed mice regardless of genotype (Supplemental Figures S5N–P, R, http://links.lww.com/HC9/C69). Notably, hepatic p-JNK was significantly downregulated in adipocyte-specific gp130 KO mice (Supplemental Figure S5Q, http://links.lww.com/HC9/C69). These findings suggest that KD intervention does not alter key mediators of lipolysis in response to glucose treatment.

Glucose injection inhibits lipolysis by increasing insulin levels, whereas lower insulin levels in the absence of glucose enhance lipolysis, providing insights into lipid metabolism under low insulin conditions. Therefore, to investigate the effects of KD on glucose and lipid metabolism, gp130 KO and control mice were dissected without glucose injection. Unlike glucose-injected mice, KD significantly reduced p-HSL levels independent of genotype in inguinal white adipose tissue, and p-AKT levels were unchanged (Figures 5B–D). In epiWAT, p-HSL levels were significantly reduced in KD-fed mice compared with chow feeding, regardless of genotype (Figures 5E, F), aligning with the reduction of inguinal white adipose tissue p-HSL levels (Figures 5B, C). Notably, epiWAT p-HSL protein levels were significantly decreased in KD-fed gp130^∆adipo^ mice compared with controls (Figures 5E, F). Similar p-AKT protein levels observed in epiWAT across diets and genotypes (Figures 5E, G) suggest that adipocyte gp130 does not directly regulate insulin sensitivity.

Next, we examined the effects of KD on liver lipophagy and stress pathways in adipocyte-specific gp130 knockout mice. After 3 days of KD, markers of lipophagy, including ATG12, p62, and LC3, showed no differences (Figure 5H). We further examined the hepatic phenotype by analyzing the activation of JNK and p38 MAPK stress kinases in adipocyte-specific gp130 KO mice. These stress kinases are activated by FFA, oxidative stress, and inflammation in obesity, aggravating insulin resistance and hepatic lipid accumulation, contributing to MASLD.^38^,^39^,^40^​ Notably, liver p-JNK and p-p38 MAPK activation were significantly downregulated in KD-fed gp130^∆adipo^ mice compared with controls (Figures 5H–J). Collectively, these findings reveal that reduced hepatic steatosis in KD-fed gp130^∆adipo^ mice may result from blunted WAT lipolysis and, hence, reduced FFA flux to the liver, as suggested by decreased p-HSL levels in epiWAT and lower hepatic p-JNK and p-p38 MAPK protein levels. In line with these findings, hepatic expression of genes involved in lipolysis (*Atgl*, *Mgl*), FFA uptake (*Lpl*), triglyceride synthesis (*Mgat3*, *Dgat1*, *Dgat2*, *Dgkd*), fatty acid β-oxidation (*Cpt1a*), and phospholipid metabolism (*Lpin2*, *Plpp2*, *Cds2*) was not significantly altered in gp130^∆adipo^ mice compared with controls (Supplemental Figures S6A–L, http://links.lww.com/HC9/C69).

### Adipocyte-specific gp130 deletion protects against IL-6-induced hepatic lipid accumulation

**FIGURE 6:**
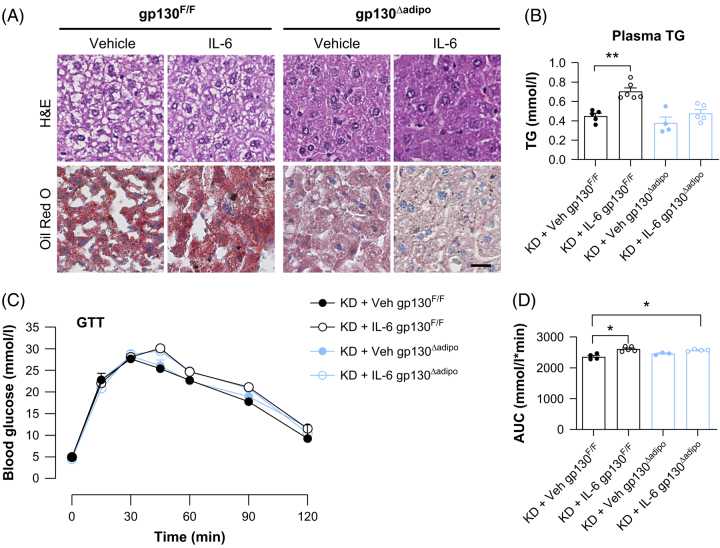
Adipocyte-specific gp130 deletion protects against IL-6–induced hepatic lipid accumulation. (A) Representative H&E and Oil Red O staining of liver sections from KD-fed control (gp130^F/F^) and adipocyte-specific gp130 knockout (gp130^Δadipo^) mice treated with recombinant IL-6 (1 mg/kg/d) or vehicle for 3 days. Scale bars: 100 µm. (B) Plasma TG levels (n = 4–5 per group). (C, D) i.p.GTT and AUC analysis in KD-fed mice treated with IL-6 or vehicle (n = 4–5 per group). Data are presented as mean ± SEM. *p<0.05, **p<0.01, by 2-way ANOVA followed by Bonferroni post hoc test. Abbreviations: i.p.GTT, Intraperitoneal glucose-tolerance test; KD, ketogenic diet; TG, triglyceride.

We next investigated whether adipocyte-specific gp130 deletion can protect the mice from IL-6-induced hepatic lipid accumulation. We administered recombinant IL-6 protein daily for 3 days to control or adipocyte-specific gp130 knockout mice during KD feeding. As expected, ketogenic feeding has resulted in hepatic steatosis in both vehicle- and IL-6-treated gp130^F/F^ mice, while gp130^∆adipo^ mice were protected from IL-6-induced steatosis (Figure 6A). Plasma triglyceride levels in control mice but not in adipocyte-specific gp130 knockout mice were significantly increased after IL-6 treatment (Figure 6B). IL-6 worsened glucose intolerance compared with vehicle-treated mice in both genotypes (Figures 6C, D). These data indicate that adipocyte gp130 deletion protects mice from KD-induced hepatic steatosis in IL-6–treated mice but not glucose intolerance.

### Adipocyte-specific gp130 deletion increases adipocyte size but reduces liver lipid accumulation under long-term KD

**FIGURE 7:**
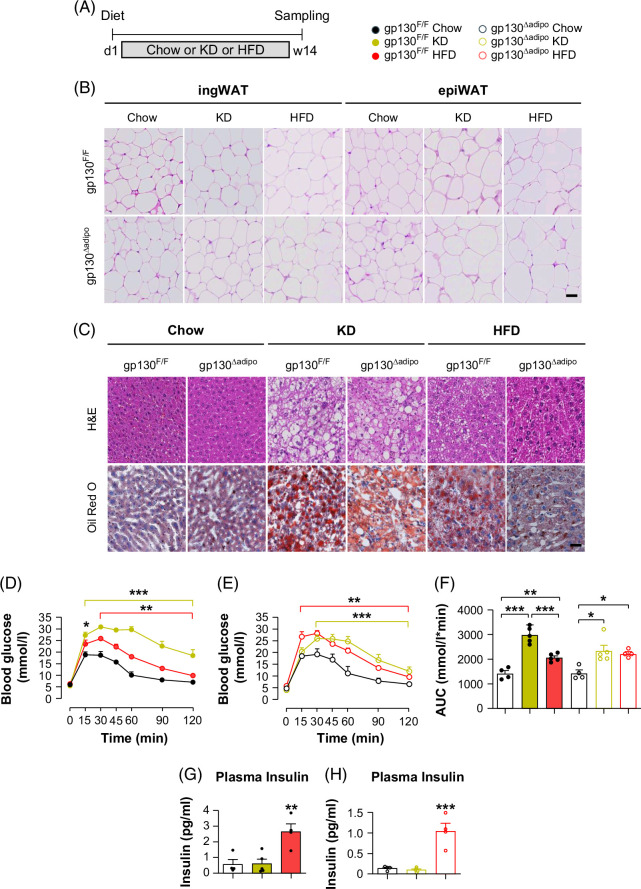
Adipocyte gp130 deletion decreases 14 weeks of KD-induced hepatic steatosis and protects from HFD-induced fatty liver. (A) Experimental scheme for a chow, HFD, or KD feeding gp130^F/F^ (WT) and gp130^∆adipo^ (KO) mice for 14 weeks and fasted for 6 hours before sampling. (B) Representative images of ingWAT and epiWAT sections stained with H&E from chow, HFD, or KD-fed gp130^F/F^ and gp130^∆adipo^ mice; scale bar represents 100 μm. (C) Representative images of liver sections stained with H&E, Oil red O from Chow, HFD, or KD-fed gp130^F/F^ and gp130^∆adipo^ mice; scale bar represents 100 μm. (D, E) i.p.GTT and (F) AUC in WT or KO mice fed chow, HFD, or KD (n=4 per group). (G, H) Plasma insulin in WT or KO mice fed chow, HFD, or KD (n=4 per group). All values are expressed as mean ± SEM. *p<0.05; **p<0.01, ***p<0.001, by 2-way ANOVA + Tukey’s multiple comparisons for D, E; 1-way ANOVA + Tukey’s multiple comparisons for F–H. Abbreviations: epiWAT, epididymal white adipose tissue; HFD, high-fat diet; ingWAT, inguinal white adipose tissue; KD, ketogenic diet.

Given that adipocyte-specific gp130 KO mice were protected from 3 days of KD-induced hepatic steatosis, we aimed to investigate the long-term effects of KD. Mice were fed chow, KD, or HFD for 14 weeks to assess the prolonged impact of KD on glucose and lipid metabolism (Figure 7A). HFD-fed mice exhibited increased body weight and fat tissue mass regardless of genotype (Supplemental Figures S7A–C, http://links.lww.com/HC9/C69). KD-fed gp130^∆adipo^ mice exhibited increased adipocyte size in epiWAT compared with chow-fed mice, while liver mass remained similar across all dietary interventions (Supplemental Figure S7C, http://links.lww.com/HC9/C69, Figure 7B). However, liver weight was significantly increased in KD-fed controls compared with chow-fed mice (Supplemental Figure S7B, http://links.lww.com/HC9/C69).

Histological examination demonstrated that long-term KD feeding increased hepatic lipid accumulation in gp130^F/F^ mice compared with chow or HFD-fed mice (Figure 7C), indicating aggravated KD-induced hepatic steatosis. Confirming previous findings,^32^ gp130^∆adipo^ mice were protected from HFD-induced liver steatosis. Similarly, gp130^∆adipo^ mice showed a trend toward reduced hepatic lipid droplet accumulation after 14 weeks of KD compared with controls (Figure 7C).

In gp130^F/F^ mice, KD and HFD-feeding induced glucose intolerance compared with chow (Figures 7D, F). Moreover, compared with HFD-feeding, KD resulted in hyperglycemia (Figures 7D, F). Adipocyte-specific gp130 deletion did not prevent KD-induced glucose intolerance but showed a trend toward improvement relative to controls (Figures 7E, F). HFD-fed mice showed higher systemic insulin levels than KD-fed mice, regardless of genotype (Figures 7G, H), suggesting HFD-induced insulin resistance. These findings indicate that KD did not increase insulin levels due to reduced carbohydrate intake and increased usage of ketone bodies as energy sources.^32^

Altogether, adipocyte-specific gp130 deletion protects against long-term HFD-induced fatty liver and shows trends toward protection from KD-induced steatosis but not diet-induced glucose intolerance.

## DISCUSSION

The molecular mechanisms underlying the pathogenesis of MASH are not yet fully explored, mainly due to a lack of animal models that accurately replicate human MASH under thermoneutral living conditions. Recently, we reported that KD feeding for 14 weeks at TN induced liver fibrosis and MASH in C57BL/6 mice through liver IL-6-JNK signaling.^32^ Our data suggest that, under thermoneutral conditions, KD-fed mice might be a useful MASH rodent model for studying MASH mechanisms. However, the absence of obesity, a key source of lipotoxicity and systemic inflammation in human MASH, is a limitation of this model and warrants further exploration.

In the current study, KD under RT induced hepatic steatosis and inflammation, but did not progress to MASH or fibrosis. Previous studies reported that thermoneutral housing enhances immune responsiveness in HFD-fed mice,^41^ whereas cold stress under standard housing inhibits immune responses.^42^ For example, deleting TLR-4 protects mice from HFD-induced MASLD at TN by modulating immune responses.^41^ In methionine choline–deficient diet-fed mice, thermoneutral housing increased hepatic inflammation and MASLD scores.^43^ These findings highlight the importance of temperature in diet-induced MASLD through inflammation.

KD promotes weight loss and improves glucose metabolism in obese patients, but its effects on the liver in healthy individuals or animal models remain unclear. In humans, KD is known to change gut microbiota and lipid profiles, increasing inflammation^44^ and cholesterol, and raising cardiovascular concerns.^45^,^46^ In mice, 2 weeks of KD altered gut microbiota, induced glucose intolerance, and hepatic steatosis by disrupting fatty acid metabolism.^47^ Our liver RNA sequencing data revealed increased *Lpl, Pparg*, and *Cd36* expressions in KD-TN versus KD-RT, suggesting enhanced lipid uptake, triglyceride storage, and accelerated fatty liver progression at TN. In line with our findings, KD activates hepatic PPARγ to boost GDF15, promoting body weight loss,^28^ while hepatocyte-specific PPARγ KO mice are protected from MASH.^36^ Furthermore, *Lpl* upregulation enhances IL-6-mediated JNK activation, driving inflammation and oxidative stress linked to MASH progression.^36^ These findings suggest a pivotal role for LPL and PPARγ in mediating KD-induced hepatic steatosis and MASLD.

Moreover, downregulation of *Ppara* and *Pgc1a* highlights mechanistic links that exacerbate KD-induced steatosis. In agreement, hepatocyte-specific ablation of *Ppara* is associated with liver inflammation and MASLD in HFD-fed mice,^35^ while mice overexpressing *Pgc1a* exhibit reduced inflammation and are protected from hepatic steatosis.^37^ Similarly, our biological process analyses showed increased inflammatory and immune responses and ECM organization in KD-TN. Network analyses also revealed upregulated inflammatory responses and JNK signaling in KD-TN, including *Tnf, Il-1b, Il-6, Ccl21, Mapk13, Tlr4*, and *Traf1.* Notably, the elevated expression of *Col1a1* and *Anxa2* in KD-TN suggests enhanced ECM deposition that may initiate fibrosis, along with increased *Nfkb2* and *Krt8*, which accelerate inflammation and cellular remodeling, key steps in the progression of steatosis to MASH. Therefore, the upregulation of *Il-6*, *Tnf, Mapk13*, *and Col1a1* pathways under KD at TN likely drives steatosis progression to MASH and fibrosis.

Based on the upregulation of IL-6 and JNK signaling identified in KD-induced MASH, we investigated whether inhibiting IL-6-gp130 signaling in the liver could rescue mice from KD-induced MASLD. However, liver-specific gp130 deletion failed to protect mice from 3 days of KD-induced hepatic steatosis or insulin resistance. Given this limited role of hepatic gp130, we investigated the impact of adipocyte-specific gp130 deletion in KD-induced metabolic dysfunction. Importantly, adipocyte-specific gp130 deletion prevented KD-induced hepatic steatosis but not glucose intolerance and insulin resistance. This effect may result from reduced lipolysis in epiWAT, suggested by lower p-HSL protein levels and larger adipocyte size, which might attenuate hepatic lipid accumulation. Supporting our findings, we previously reported that HFD-fed adipocyte gp130 KO mice exhibited reduced lipolysis in mesenteric fat, thereby protecting them from diet-induced hepatic steatosis.^25^ In HFD-fed mice, adipocyte-specific IL-6-gp130 signaling induced FFA release from visceral adipocytes, promoting obesity-induced hepatic insulin resistance and steatosis.^25^,^48^

Elevated circulating leptin levels in KD-fed adipocyte-specific gp130 KO mice may regulate lipolysis. In contrast, HFD-fed adipocyte-specific gp130 KO mice revealed similar adipocyte size but reduced circulating leptin levels.^25^,^32^,^48^ Hence, adipocyte-specific gp130 signaling may regulate adipocyte size and leptin secretion in a diet-dependent manner. Our data demonstrated that adipocyte IL-6-gp130 signaling regulates lipolysis to protect adipocyte-specific gp130 KO mice from KD-induced hepatic steatosis. Recombinant IL-6 administration further confirmed this, as IL-6–treated adipocyte-specific gp130 knockout mice were also protected from KD-induced hepatic steatosis compared with control mice.

High-cholesterol levels induce chronic inflammation, contributing to steatosis and the progression of MASLD.^32^ In addition, adipocyte IL-6-gp130 function is complex and activates the JAK/STAT, PI3K/AKT, and MAPK pathways, leading to inflammation and dysregulated lipid metabolism.^42^,^43^,^44^,^45^ Notably, stress signaling pathways such as JNK and p38 MAPK are downregulated under KD in the livers of adipocyte-specific gp130 KO mice. Decreased cholesterol levels and reduced WAT lipolysis in KD-fed adipocyte-specific gp130 KO mice may contribute to reduced hepatic p-JNK and p-p38 protein levels, indicating protection from KD-induced steatosis. These findings indicate improved liver lipid metabolism in KD-fed adipocyte-specific gp130 KO mice.

In high-cholesterol diet-fed mice, ganoderic acid decreases the expression of p-JNK, thereby ameliorating fatty liver.^49^ In addition, the inhibition of JNK rescued the mice from KD-induced hepatic steatosis and insulin resistance by decreasing cholesterol levels.^32^ Meanwhile, the activation of p38 MAPK increases lipolysis by upregulating p-HSL, thereby contributing to the development of hepatic steatosis.^50^ Our data suggest that KD feeding in adipocyte-specific gp130 KO mice promotes fat storage in epiWAT, potentially reducing lipolysis and preventing KD-induced hepatic steatosis by downregulating p-HSL, p-JNK, and p-p38 pathways.

All experiments of the present study were conducted in male mice, which may not fully capture sex-specific metabolic responses. Further studies are warranted to test the direct effects of *Tnf* and *Mapk13* in driving fibrosis and MASH at TN.

In summary, our data indicate that KD feeding induces fibrosis and MASH through upregulated *Il-6*, *Tnf*, and *Mapk13* pathways at TN, whereas adipocyte-specific gp130 deletion prevents KD-induced hepatic steatosis through reduced p-HSL, p-JNK, and p-p38 MAPK signaling. Furthermore, after pharmacological activation of IL-6, gp130 KO mice remained protected from KD-induced MASLD. These findings highlight the adipocyte IL-6-gp130 axis as a therapeutic target for MASLD progression.

## Supplementary Material
