# Supplementary material for: Adipocyte-specific deletion of gp130 prevents ketogenic diet–induced hepatic steatosis
Source: Hepatol Commun. 2025 Aug 26;9(9):e0782. doi: 10.1097/HC9.0000000000000782 (PMC12385040; doi:10.1097/HC9.0000000000000782)
Supplement: Supplementary file 1 [file hc9-9-e0782-s001.pdf]

Adipocyte-specific deletion of gp130 prevents ketogenic diet-induced hepatic steatosis

Berkay Senkalfa[1], Melanie Gloor[1], Ronja Podlaszewski[1], Revati S. Dewal[1], Carla Horvath[1], Vissarion Efthymiou[1], Adhideb Ghosh[1], Stephan Wueest[2,3], Daniel Konrad[2,3,4], Christian Wolfrum[1*], Tenagne D. Challa[1*]

Affiliations:

[1]Institute of Food Nutrition and Health and Department of Health Sciences and Technology, Eidgenössische Technische Hochschule Zürich (ETH), CH-8603 Schwerzenbach, Switzerland

[2]Division of Pediatric Endocrinology and Diabetology, University Children's Hospital, University of Zurich, CH-8008 Zurich, Switzerland

[3]Children's Research Center, University Children's Hospital, University of Zurich, CH-8008 Zurich, Switzerland

[4]Zurich Center for Integrative Human Physiology, University of Zurich, CH-8057 Zurich, Switzerland

Author contributions:

BS, TDC, and CW wrote the manuscript. TDC and CW jointly supervised this work. TDC, BS, and CW designed experiments and interoperated the data. MG, BS, RP, and TDC performed the experiments. BS, TDC, SW, DK, and CW interpreted the data and revised the manuscript. RSD, CH, VE, and AG helped with project coordination. BS, AG, and TDC helped with the analysis of RNA sequencing data.

Address for correspondence: Dr. Tenagne D. Challa, Dr. Christian Wolfrum

Address: Eidgenössische Technische Hochschule Zürich (ETH, Zürich). Department of Health Sciences and Technology. Schorenstrasse 16, CH-8603 Schwerzenbach, Switzerland.

Tel +41 44 65574 64, E-Mail: **tenagne.challa@hest.ethz.ch** (Dr. Tenagne D. Challa)

Tel +41 44 655 74 5, E-Mail: **christian-wolfrum@ethz.ch** (Prof. Dr. Christian Wolfrum)

Conflict of Interest: The authors declare no competing interests.

Keywords: Ketogenic diet, MAFLD, MASH, gp130, IL-6, HSL, Lipolysis, JNK, p38, WAT

List of Abbreviatons:

MAFLD: metabolic-associated fatty liver disease
T2D: type 2 diabetes
MASH: metabolic-associated steatohepatitis
KD: ketogenic diet
MD: mediterranean diet
HFD: high-fat diet
FFA: free fatty acid
RT: room temperature
TN: thermoneutrality
iBAT: interscapular brown adipose tissue
epiWAT: epididymal white adipose tissue
mesWAT: mesenteric white adipose tissue
ingWAT: inguinal white adipose tissue

Supplemental Digital Content

1. Supplementary materials and methods

1.1. Glucose tolerance test

To investigate whether the KD effect on glucose tolerance is acute or progressive 12-week-old male mice were randomly assigned to either chow, KD, or HFD for 3 or 7 days or 14 weeks. Before testing, mice were fasted for 6h, and baseline glucose levels were measured. After that, glucose (2 g/kg body weight (D-glucose, Sigma in 0.9% saline)) was injected intraperitoneal (i.p.) or orally and blood glucose concentration was measured from tail-tip blood after 15, 30, 45, 60, 90, and 120 min by using a glucometer (Accu-Check Aviva glucose strip system, #07400918016, #06453988016; Roche Diagnostics International, Basel, Switzerland).

1.2. Tissue lysate and plasma parameters

The mice were fasted for 6 hours and subsequently injected with or without glucose (2 g/kg body weight, i.p) 30 minutes before tissue and blood sampling. Circulating insulin concentration was measured using an ultra-sensitive mouse insulin ELISA kit (Crystal Chem, #90080), and leptin was measured using a Leptin ELISA Kit (Crystal Chem, #80968). Plasma cytokine interleukin 6 (IL-6) was measured using MSD technology (Meso Scale Discovery, Gaithersburg, MD, USA). FFA was measured using Non-Esterified Fatty Acids (NEFA) measurement kit (Fujifilm, #436-91995) and TG was determined using triglycerides-LQ (Spinreact, #41031). Plasma Cholesterol and Liver cholesterol were measured by Cobas Roche (Hitachi Kit #11877771, Roche Diagnostics International). All analysis was carried out according to the manufacturer's protocols.

1.3. Protein extraction and Western Blot

Equal amounts of protein (20-60μg) were resolved on (8-15%) a SDS-polyacrylamide gel (PAGE) gel and transferred onto a nitrocellulose membrane (#1620112, Bio-Rad). Membranes were blocked for 1 hour at room temperature with 5% milk in TBS-T, then incubated overnight with primary antibodies. The following primary antibodies were used: anti-phospho-AKT (#13038; diluted 1:2000),

anti-AKT (#9272; diluted 1:2000), anti-phospho-SAPK/JNK (Thr183/Tyr185, #4668; diluted 1:2000), anti-JNK (#9252; diluted 1:2000), anit-phospho-p38 MAPK (Thr180/Tyr182) (#4511S; diluted 1:1000), anit-p38 MAPK (#8690S; diluted 1:1000), anit-ATG12(abcam, #ab303488; diluted 1:1000), anit-LC3B (#2775; diluted 1:50), anti-SQSTM1/p62 (#5114S; diluted 1:1000), anti-phospho-HSL (#45804, 1:1000), anti-HSL (#4107, 1:1000), anti-ATGL (#2138, 1:1000), anti-perilipin-1 XP (#9349), anti-y-tubulin (Sigma, #T6557), anti-HSP90 (#4877) and anti-GAPDH (#2118, 1:1000). If not mentioned differently, all antibodies were obtained from Cell Signaling Technology). Next, they were washed and incubated with corresponding secondary HRP–conjugated antibodies (1:10.000, Calbiochem): anti-rabbit IgG (1:4000, Millipore, #401392) or anti-mouse IgG (1:4000, Millipore #401253). The protein signals were detected by an LAS 4000 mini–Image Quant system (GE Healthcare Life Sciences) and quantified with Image Lab (version 6, from biorad).

1.4. Histological analysis

Histological analysis was conducted as described in our recent work[6], with modifications for fat tissue staining. Briefly, 12-week-old male mice were assigned to chow, KD, or HFD for 3, 7 days, or 14 weeks at room RT. Liver and fat tissues were fixed in 4% paraformaldehyde (PBS, pH 7.4, Gibco) for 24 hours at 4°C, then dehydrated and embedded in paraffin. Fat tissue was stained with hematoxylin and eosin (H&E). Liver sections (3µm) were stained with H&E, Oil Red O, Granular osmiophilic material (GOM)/Masson's trichrome, and cleaved caspase-3. Prof. Dr. Anja Kipar (Pathologist, Zurich University) performed tissue processing and staining using automated strainers. Slides were scanned and analyzed digitally in blind conditions. For quantitative evaluation, the NASH Clinical Research Network Scoring System[9], and murine NAFLD activity scores were applied to assess inflammation, steatosis, hepatocyte ballooning, fibrosis, NASH, and MAFLD[8,9].

1.5. Quantification and statistical analysis

A power calculation was performed based on the results of previous work by our group to calculate animal numbers[8]. All data are expressed as mean ± standard error of the mean. The significance

was determined using a two-tailed, unpaired Student's *t*-test, one-way ANOVA with Newman–Keuls correction for multiple group comparisons, or two-way ANOVA with Bonferroni multiple comparisons/Tukey's multiple comparison. Statistical tests were calculated using GraphPad Prism 10.3.1(GraphPad Software, San Diego, USA). *P*-values < 0.05 were considered significant.

1.6. DATA AND CODE AVAILABILITY

Bulk RNAseq data is available in GEO under the accession number *GSE294746*. The original data for the uncropped and unmodified images in this paper are available upon request.

Supplementary Figure Legends

Supp. Figure 1. KD increases Inflammatory pathways and induces NASH and fibrosis. Chow, HFD, or KD-fed C57BL/6N mice at thermoneutrality (TN) or room temperature (RT) for 14 weeks, fasted for 6 hours, and were injected with glucose (2 g/kg body) 30 min before sampling. RNA sequencing (RNA-seq) was performed on mice liver (n=4 per group). **(A-G)** Volcano plot analyses, (**E-I)** Heat map of differentially expressed genes. **(J)** Volcano plot analyses. Differentially expressed genes were analyzed at the threshold of p <0.01, log2 ratio> 0.5, and fold change of log2 ratio> 0.5. Data are presented as the regularized (r) log of the counts.

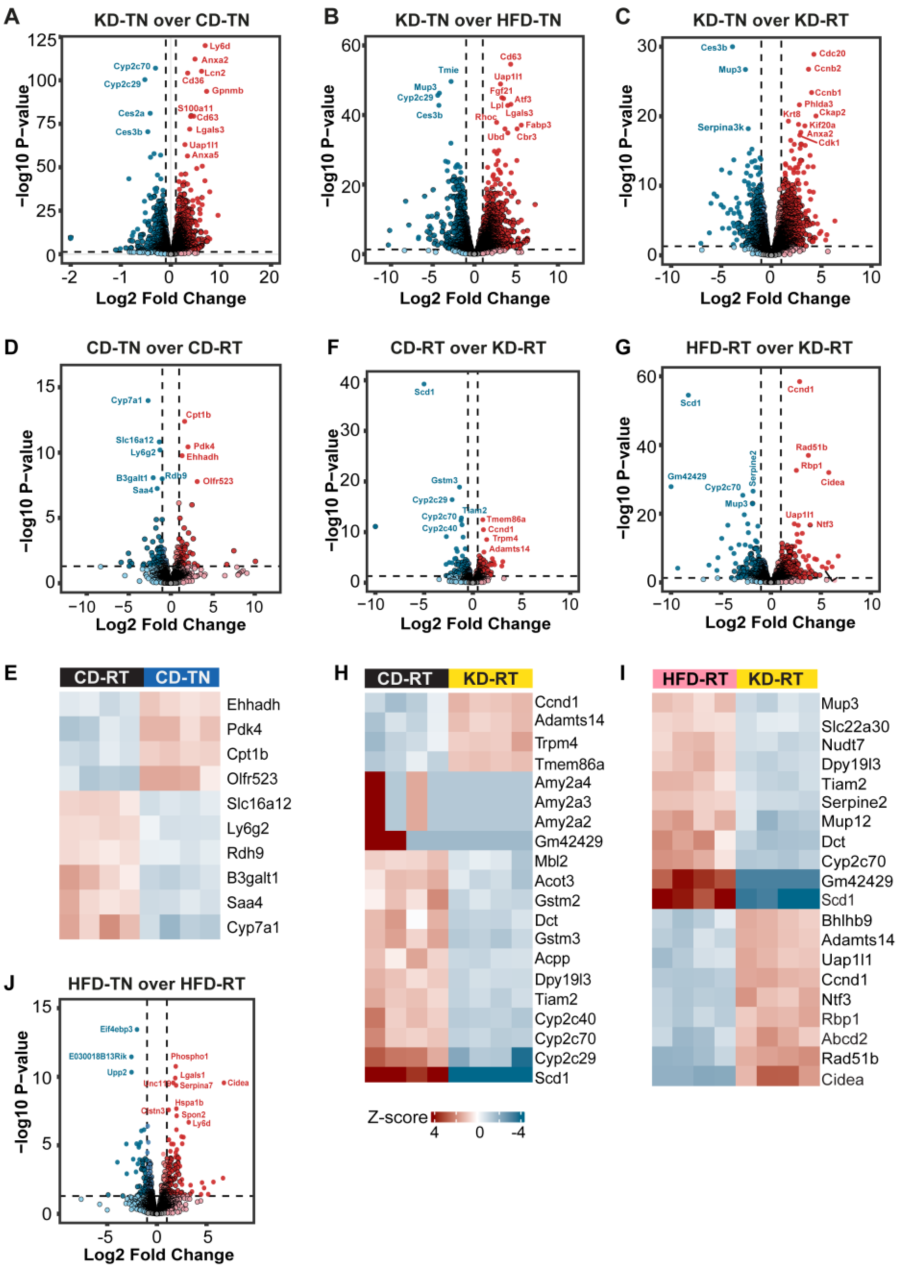

Supplementary Figure 1

Supp. Figure 2. Expression of Lipolytic and Lipogenic Pathways at mRNA in KD-fed C57BL/6N Mice. A) The experimental scheme for chow, HFD, or KD feeding C57BL/6N mice at room temperature (RT) for 14 weeks, fasted for 6 hours, and injected with glucose (2 g/kg body) 30 min before sampling. **(B-P)** Investigation and validation of select genes identified in RNAseq data by qPCR, (n05-7). Values are presented as mean ± SEM. *(P<0.05), **(P<0.01), ***(P<0.001), by one-way ANOVA + Tukey's multiple comparisons.

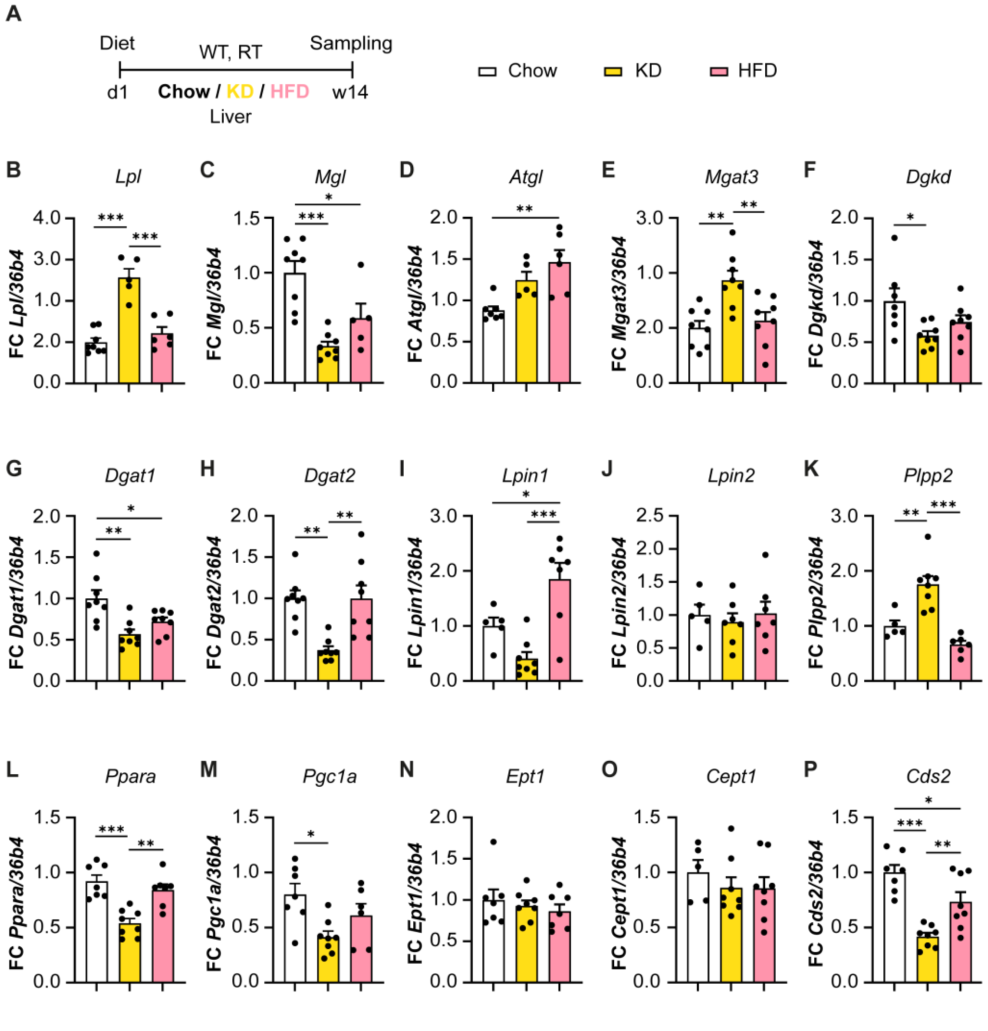

Supplementary Figure 2

Supp. Figure 3. Liver-specific gp130 deletion does not protect mice from KD-induced glucose intolerance and hepatic steatosis. Chow or KD feeding WT ($gp130^{F/F}$) and liver gp130 KO ($gp130^{\Delta alb}$) mice for 3 days fasted for 6-hrs before sampling. (**A**) Gp130 expression in intrascapular brown adipose tissue (iBAT), inguinal white adipose tissue (ingWAT), epididymal fat (epiWAT), and liver, n=6 per group. (**B**) Body weight measurement (n=5 per group). (**C-H**) Representative western blots analyses of ATGL, p-HSL, perilipin, p-JNK, and p-AKT protein expression and quantification in epiWAT (n=5 per group). (**I-N**) Representative western blots analyses of ATGL, p-HSL, perilipin, p-JNK, and p-AKT protein expression and quantification in mesenteric fat (n=5 per group). Values are presented as mean ± SEM. ***(P < 0.001) by Student's t-test.

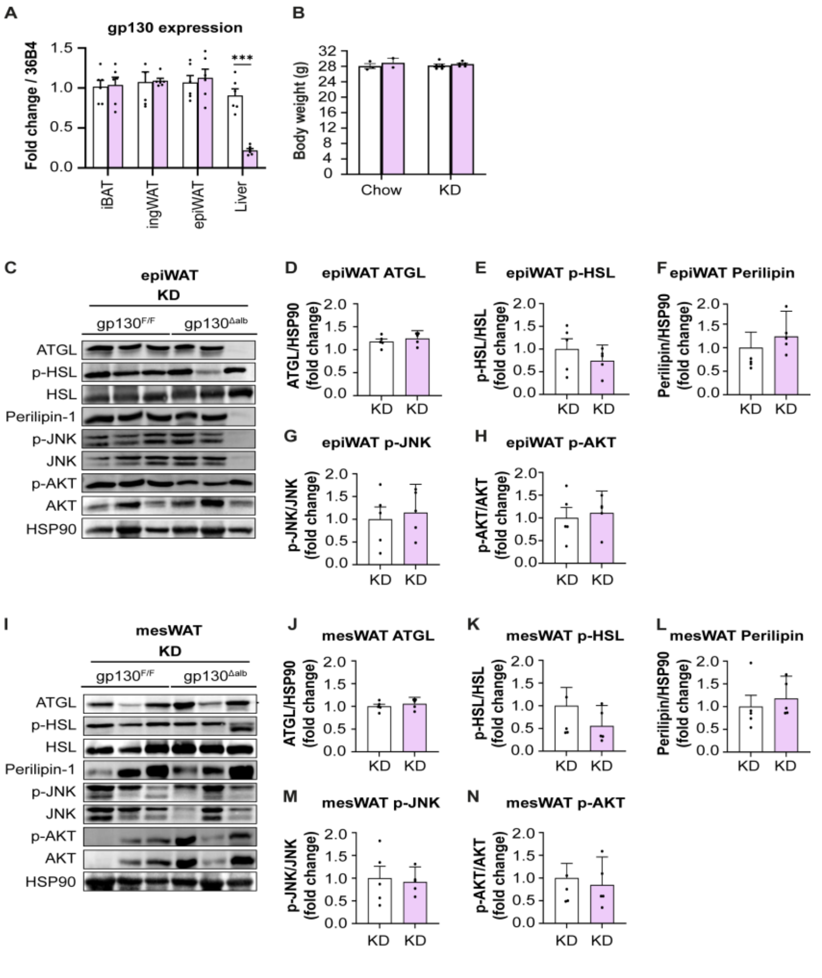

Supplementary Figure 3

Supp. Figure 4. (A-B), Insulin tolerance test (ITT) in gp130$^{F/F}$ and gp130$^{\Delta adipo}$ mice after 3 days of ketogenic diet feeding. Blood glucose levels and the area under the curve (AUC) are presented as mean ± SEM, with n = 5–6 per group.

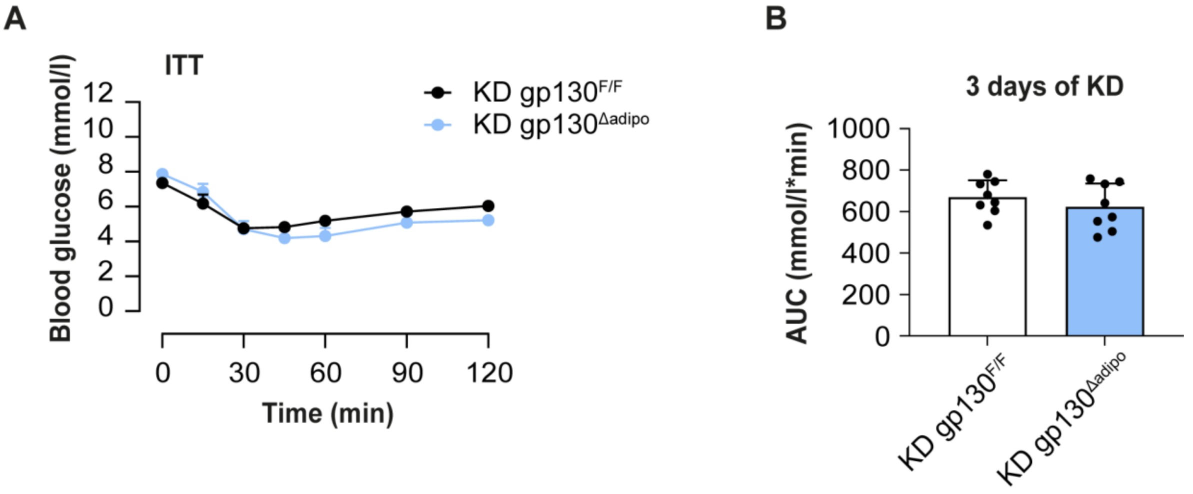

Supplementary Figure 4

Supp. Figure 5. Effects of KD in gp130 adipocytes KO mice. Insulin signaling and lipolytic markers in adipose tissues and the liver were assessed after glucose injection to evaluate insulin-mediated effects. **(A)** Experimental scheme for KD feeding gp130$^{F/F}$ (WT) and gp130$^{\Delta adipo}$ (KO) mice for 3 days, then injected with glucose (2 g/kg body) and fasted for 6-hrs before sampling. (**B-G**) Representative western blots analyses of ATGL, p-HSL, perilipin, p-JNK, and p-AKT protein expression and quantification in epiWAT (n=4 per group). (**H-M**) Representative western blots analyses ATGL, p-HSL, perilipin, p-JNK, and p-AKT protein expression and quantification in mesenteric WAT (mesWAT) (n=4 per group). (**N-R**) Representative western blots analyses of ATGL, p-HSL, p-JNK, and p-AKT protein expression and quantification in liver (mesWAT) (n=4 per group). All values are expressed as mean ± SEM. **p<0.01, by Student's t-test.

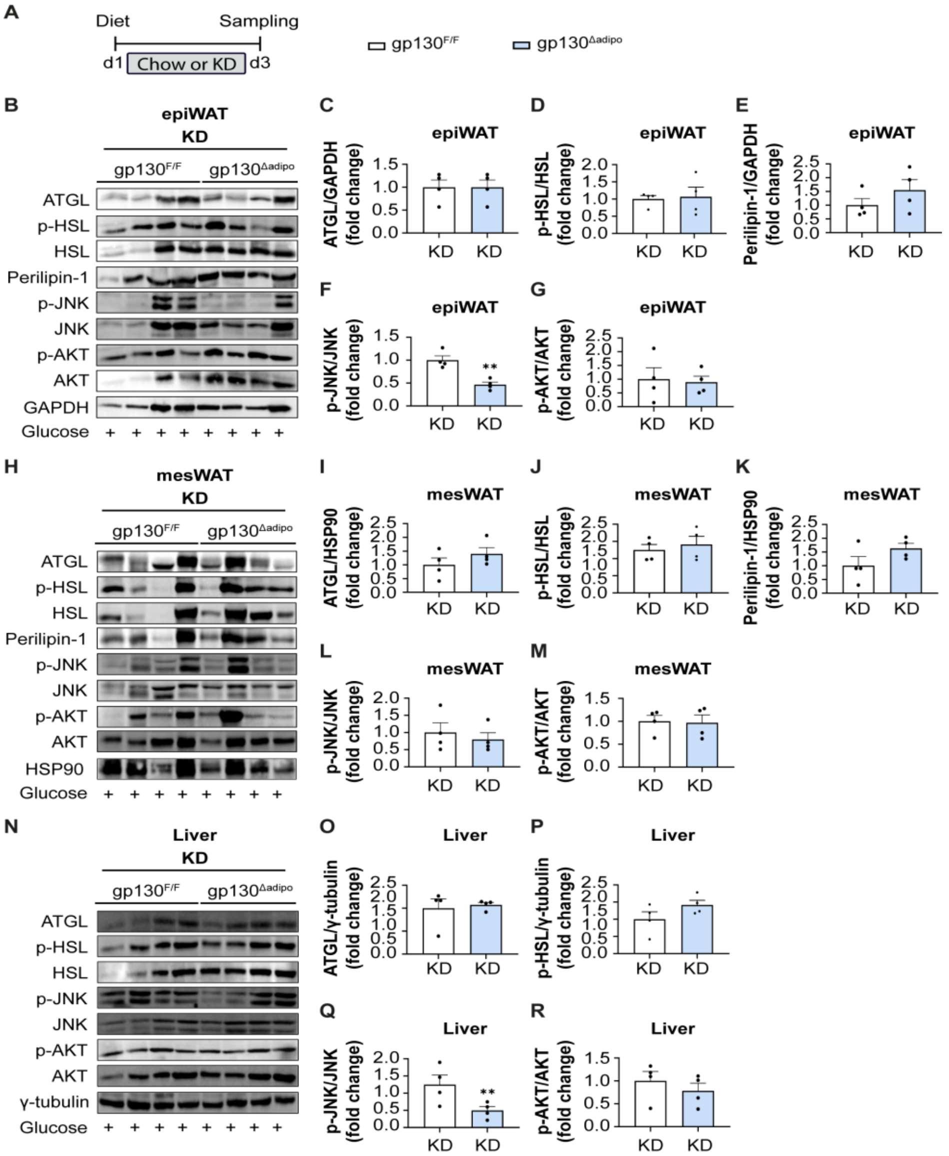

Supplementary Figure 5

Supp. Figure 6. Liver mRNA expression (A-L) of genes involved in lipolysis (*Mgl*, *Atgl*), free fatty acid uptake (*Lpl*), triglyceride synthesis (*Mgat3*, *Dgat1*, *Dgat2*, *Dgkd*), phospholipid metabolism (*Lpin2*, *Ept1*, *Plpp2*, *Cds2*, *Cept1*), and fatty acid β-oxidation (*Cpt1a*) in gp130$^{F/F}$ and gp130$^{\Delta adipo}$ mice after 3 days of ketogenic diet feeding. Data are presented as mean ± SEM. n = 5–6 mice per group.

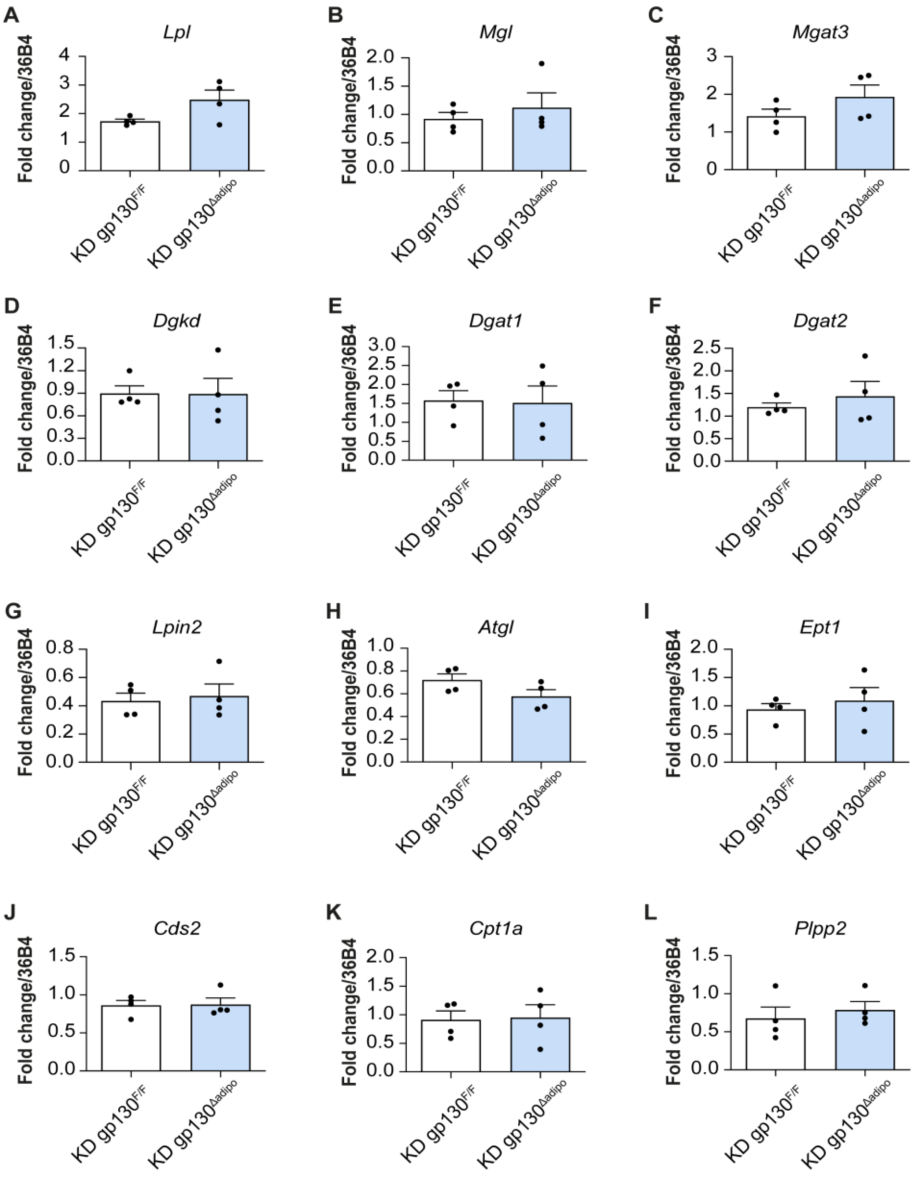

Supplementary Figure 6

Supp. Figure 7. Chow or KD feeding showed similar body weight changes in adipocyte gp130 KO mice. A chow, HFD, or KD feeding gp130$^{F/F}$ (WT) and gp130$^{\Delta adipo}$ (KO) mice for 14 weeks and fasted for 6 hours before sampling. **(A)** Body weight, **(B)** Fat and liver mass in WT mice. **(C)** Fat and liver mass in KO mice (n=4 per group). All values are expressed as mean ± SEM. *(P<0.05), ***(P<0.001), by one-way ANOVA + Tukey's multiple comparisons.

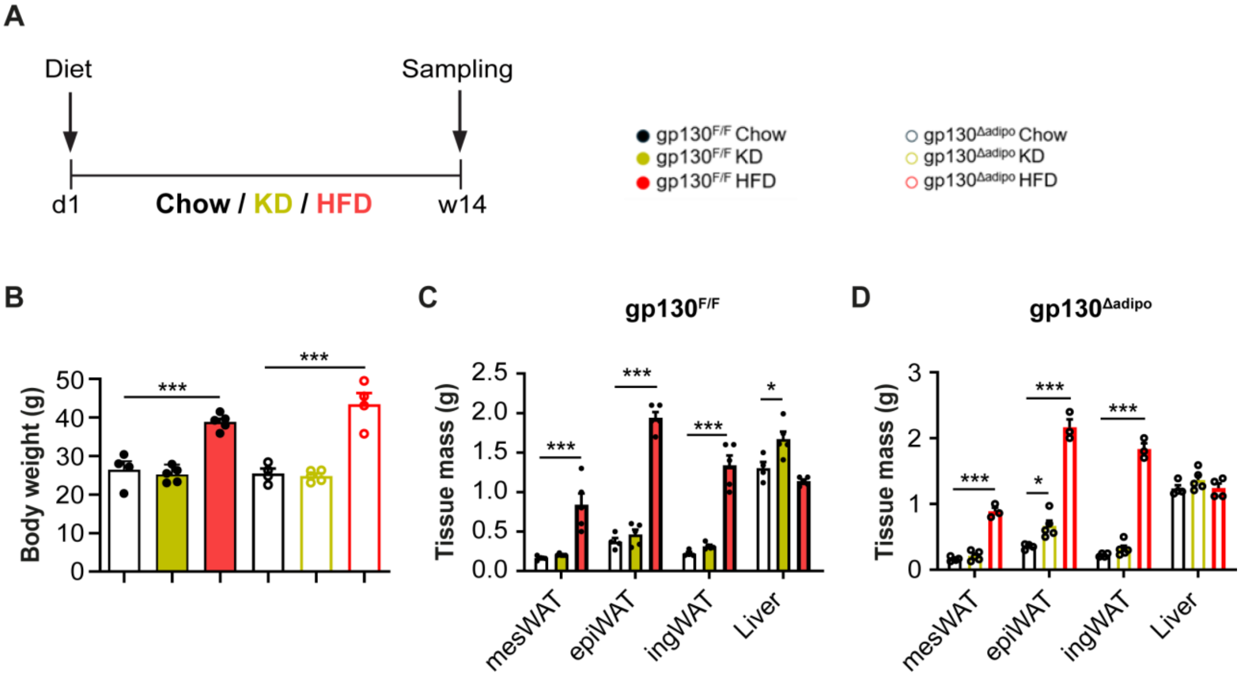

Supplementary Figure 7

Supplementary References

1. Moran-Costoya A, Proenza AM, Gianotti M, et al. Sex differences in nonalcoholic fatty liver disease: estrogen influence on the liver-adipose tissue crosstalk. *Antioxidants & Redox Signaling.* 2021;35(9). doi.org/10.1089/ars.2021.0044

2. Gonzalez-Granillo M, Savva C, Li X, et al. Selective estrogen receptor (ER)β activation provokes a redistribution of fat mass and modifies hepatic triglyceride composition in obese male mice. *Molecular and Cellular Endo.* 2020;502:110672. doi.org/10.1016/j.mce.2019.110672

3. Balaz M, Becker AS, Balazova L, et al. Inhibition of Mevalonate Pathway Prevents Adipocyte Browning in Mice and Men by Affecting Protein Prenylation. *Cell Metab*. 2019;29(4):901-

916.e8. doi:10.1016/j.cmet.2018.11.017

4. Bolger AM, Lohse M, Usadel B. Trimmomatic: a flexible trimmer for Illumina sequence data. *Bioinformatics*. 2014;30(15):2114-2120. doi:10.1093/bioinformatics/btu170

5. Bray NL, Pimentel H, Melsted P, Pachter L. Near-optimal probabilistic RNA-seq quantification. *Nat Biotechnol*. 2016;34(5):525-527. doi:10.1038/nbt.3519

6. Long F, Bhatti MR, Kellenberger A, et al. A low-carbohydrate diet induces hepatic insulin resistance and metabolic associated fatty liver disease in mice. *Molecular Metabolism*. Published online January 2023:101675. doi:10.1016/j.molmet.2023.101675

7. Kleiner DE, Brunt EM, Van Natta M, et al. Design and validation of a histological scoring system for nonalcoholic fatty liver disease. *Hepatology*. 2005;41(6):1313-1321. doi:10.1002/hep.20701

8. Clapper JR, Hendricks MD, Gu G, et al. Diet-induced mouse model of fatty liver disease and nonalcoholic steatohepatitis reflecting clinical disease progression and methods of assessment. *Am J Physiol Gastrointest Liver Physiol*. 2013;305(7):G483-495. doi:10.1152/ajpgi.00079.2013

9. Liang W, Menke AL, Driessen A, et al. Establishment of a general NAFLD scoring system for rodent models and comparison to human liver pathology. *PLoS One*. 2014;9(12):e115922. doi:10.1371/journal.pone.0115922
